# Stage and tissue-specific prognostic impact of miR-182 in NSCLC

**DOI:** 10.1186/1471-2407-14-138

**Published:** 2014-02-27

**Authors:** Helge Stenvold, Tom Donnem, Sigve Andersen, Samer Al-Saad, Lill-Tove Busund, Roy M Bremnes

**Affiliations:** 1 Institute of Clinical Medicine, University of Tromso, Tromso, Norway; 2 Department of Oncology, University Hospital of North Norway, Tromso 9038, Norway; 3 Institute of Medical Biology, University of Tromso, Tromso, Norway; 4 Department of Clinical Pathology, University Hospital of North Norway, Tromso, Norway

**Keywords:** NSCLC, Stage I-IIIA, Survival, Prognostic impact, miR-182, miRNA

## Abstract

**Background:**

MicroRNA (miR)-182 is frequently upregulated in cancers, has generally been viewed as an oncogene and is possibly connected to angiogenesis. We aimed to explore what impact miR-182 has in non-small cell lung cancer (NSCLC), and more explicitly its correlation with angiogenic markers.

**Methods:**

From 335 unselected stage I to IIIA NSCLC carcinomas, duplicate tumor and tumor-associated stromal cores were collected in tissue microarray blocks (TMAs). *In situ* hybridization (ISH) was used to detect the expression of miR-182 in tumor cells, and immunohistochemistry (IHC) was used to detect the expression of angiogenesis related protein markers.

**Results:**

In univariate analyses, high tumor cell expression of miR-182 was a positive prognostic factor for patients with squamous cell carcinoma (SCC, P = 0.042) and stage II patients (P = 0.003). Also in the multivariate analysis, high tumor cell miR-182 expression was associated with a good prognosis in the same groups (SCC: HR 0.57, CI 95% 0.33-0.99, P = 0.048; stage II: HR 0.50, CI 95% 0.28-0.90, P = 0.020). We found significant correlations between miR-182 and the angiogenesis related markers FGF2, HIF2α and MMP-7.

**Conclusion:**

In patients with SCC and in stage II patients, high tumor cell miR-182 expression is an independent positive prognostic factor.

## Background

Lung cancer is, despite a small decline in mortality recent years, still the number one killer among cancers [1]. Non-small cell lung cancer (NSCLC) accounts for 80–85% of all lung cancers. Optimization of treatment with better surgery, cytotoxic agents and radiation therapy has not altered the prognosis much. We are now in an era where personalized medicine and targeted therapies may give new hope for this patient group [2,3]. Identification of novel molecular markers which can improve diagnosis and prognostic stratification and serve as possible therapeutic targets will be of great importance in the near future.

MicroRNAs (miRNAs) are small non-coding nucleotides. They post-transcriptionally control the stability and translation of mRNAs. Today, we know more than 1500 different miRNAs, and each miRNA can regulate several genes [4]. Many miRNAs are located at sites of the genome known to be altered in cancers, and are frequently up- or down regulated [5]. The differences in miRNA expression between cancers make it possible to develop specific miRNA profiles for different cancer types [6].

miR-182 is one of the miRNAs often seen up-regulated in cancers. Also in NSCLC, several studies have reported miR-182 to be up-regulated, and it is generally regarded as an oncogene [7-11]. However, results are conflicting concerning its role as an oncogene or tumor suppressor. In NSCLC and other malignancies, high miR-182 expression has been associated with cell migration, metastatic properties of cancer cells and poor survival [11-13]. Recent studies have, however, found miR-182 to suppress lung cancer cell proliferation and growth of melanoma cells [14-16].

In a recent study, we screened tumor tissues from 10 worst and 10 best prognosis NSCLC cases as well as 10 normal lungs for the expression of several angiogenesis-related miRNAs [17]. miR-182 was the only miRNA among 281 tested to be up-regulated in all three comparisons: worst prognosis versus normal lung, best prognosis versus normal lung and worst prognosis versus best prognosis [17]. Besides, miR-182 appeared to be connected to angiogenesis according to the Gene Set Enrichment Analyses (GSEA) [17].

Based on these pilot data, we have explored the impact of miR-182 in our large unselected cohort of 335 NSCLC cases. *In situ* hybridization was performed on tissue micro array slides for high-throughput exploration of miR-182’s prognostic impact. Since it is known that miRNAs are highly tissue- and stage specific and miR-182, in particular, possibly connected to angiogenesis according to the GSEA, we aimed to explore 1) the prognostic impact of miR-182 also in the NSCLC subgroups and 2) its association with relevant angiogenic and hypoxia molecular markers.

## Methods

### Patients and clinical samples

Between 1990 and 2004, 371 patients with pathological stage I to IIIA non-small cell lung cancer were diagnosed at the University Hospital of North Norway and Nordland Central Hospital and treated with curative intent. Resected tissues from the primary tumors in these patients were used in our retrospective study. Out of 371 patients, 36 were excluded from the study due to radiotherapy or chemotherapy prior to surgery (n = 10), other malignancy within 5 years before NSCLC diagnosis (n = 13) or inadequate paraffin-embedded fixed tissue blocks (n = 13). Adjuvant chemotherapy was not introduced in Norway during this period (1990 – 2004). Thus, 335 patients with complete demographic and clinicopathological data were eligible for this study. Of these, postoperative radiotherapy was offered to 55 patients with non-radical surgical margins or mediastinal lymph node disease (N2).

This report includes follow-up data as of January 10, 2011. The median follow-up time of survivors was 105 months (range 73–234). Formalin-fixed, paraffin-embedded tumor specimens were obtained from the archives of the Departments of Clinical Pathology at the University Hospital of North Norway and Nordland Central Hospital. The pathological data were revised according to the 7^th^ edition of UICC TNM classification of lung cancer [18]. If the morphological characteristics for adeno- and squamous cell carcinomas were easily recognizable, it was not always necessary to do further examinations (IHC) of the tumor samples. If the tumors were not well differentiated, IHC was necessary. CK7, TTF1, p63 and CK5/6 was the markers most frequently used. The National Data Inspection Board and the Regional Ethics Committee North (REC North) approved this study.

### Microarray construction

We used a 0.6 mm-diameter stylet to sample two cores with neoplastic tissue and two cores with tumor stroma from different areas of the primary tumors from each patient. Normal lung tissue localized distant from the tumor and lung tissue sample from 20 patients without cancer diagnosis were used as controls. The TMAs were assembled using a tissue-arraying instrument (Beecher Instruments, Silver Springs, Md). Eight tissue microarray blocks were made to include all the tissue samples. Multiple 4-μm-sections were cut with a Micron microtome (HM355S) and stained by specific antibodies for immunohistochemical analyses or stained by *in situ* hybridization. The detailed methodology has been previously reported [19].

### In situ hybridization (ISH)

In situ hybridization was performed following the protocol developed by Exiqon, Vedbaek, Denmark [20]. Digoxigenin (DIG) labelled locked nucleic acid (LNA) modified probes from Exiqon for miR-182 (hsa-miR-182), positive control (U6, hsa/mmu/rno) and negative control (scramble-miR) were used in this study. Some adjustments were done to get a specific and sensitive detection of miRNA in our sections from formalin-fixed paraffin-embedded (FFPE) TMA blocks.

We placed 4 μm sections of the TMA blocks in a heater at 59˚C over night to attach cores to Super Frost Plus slides. Sections were deparaffinised with xylene (3 × 5 min) and then rehydrated with ethanol solutions (99.9% - 96% - 70%) ending up in PBS, pH 7.4. Proteinase-K (20 μg/ml) (Exiqon, Vedbaek, Denmark) treatment was done in PK-buffer (5 mM Tris–HCl, pH 7.5, 1 mM EDTA, 1 mM NaCl, autoclaved) at 37˚C for 20 min in a HYBrite automated hybridizer (Abbot laboratories, IL, US). After a PBS wash the sections were dehydrated through increasing gradient of ethanol solutions and air-dried. The LNA-probes were denatured by heating to 90˚C for 4 min. Hybridization of the LNA-probe miR-182 (100nM) and scramble miR (50nM) control was carried out in the HYBrite automated hybridizer at 50˚C for 60 min. The positive control U6 (1nM) was hybridized at 55˚C for 60 min. Stringent washes was performed in pre-heated SSC buffers, 1 × 5 min in 5x SSC, 2 × 5 min in 1× SSC and 0,2× SSC. Sections were blocked against unspecific binding in blocking solution from DIG wash and Block Buffer set (Roche, Mannheim, Germany) for 15 min at room temperature (RT). Alkaline phosphatase (AP)-conjugated anti-DIG (Roche) 1:800 was incubated for 60 min at RT for immunologic detection. After PBS-T wash the substrate enzymatic reaction was carried out with NBT/BCIP (Roche) at 30˚C in the hybridizer for 120 min. The reaction was stopped with a 2 × 5 min wash in KTBT buffer (50 mM Tris–HCl, 150 mM NaCl, 10 mM KCl). Sections were counter stained with nuclear fast red (WALDECK, ZE-012-250) at RT for 1 min and then rinsed in tap water. Dehydration followed through increasing gradient of ethanol solutions and finally mounting with Histokitt mounting medium (Assistant-Histokitt, 1025/250).

### Immunohistochemistry (IHC)

We used data from previous publications with the following antibodies for correlation analyses: VEGF (−A, –C, -D, R-1, R-2, R-3), PDGF (−A, -B, –C, -D, R-α, R-β), FGF (−2, R-1), Notch (−1, -2), Jagged1, DLL4, Hif (−1α, -2α), GLUT-1, LDH5, CAIX, PHD (−1, -2, -3), FIH, Ang (−1, -2, -4), Tie-2 and MMP (−2, -7, -9). Detailed IHC procedures for the antibodies which correlated significantly with miR-182 (FGF2, Hif2α and MMP-7) have been previously published [21-23].

### Scoring of ISH

**Figure 1:**
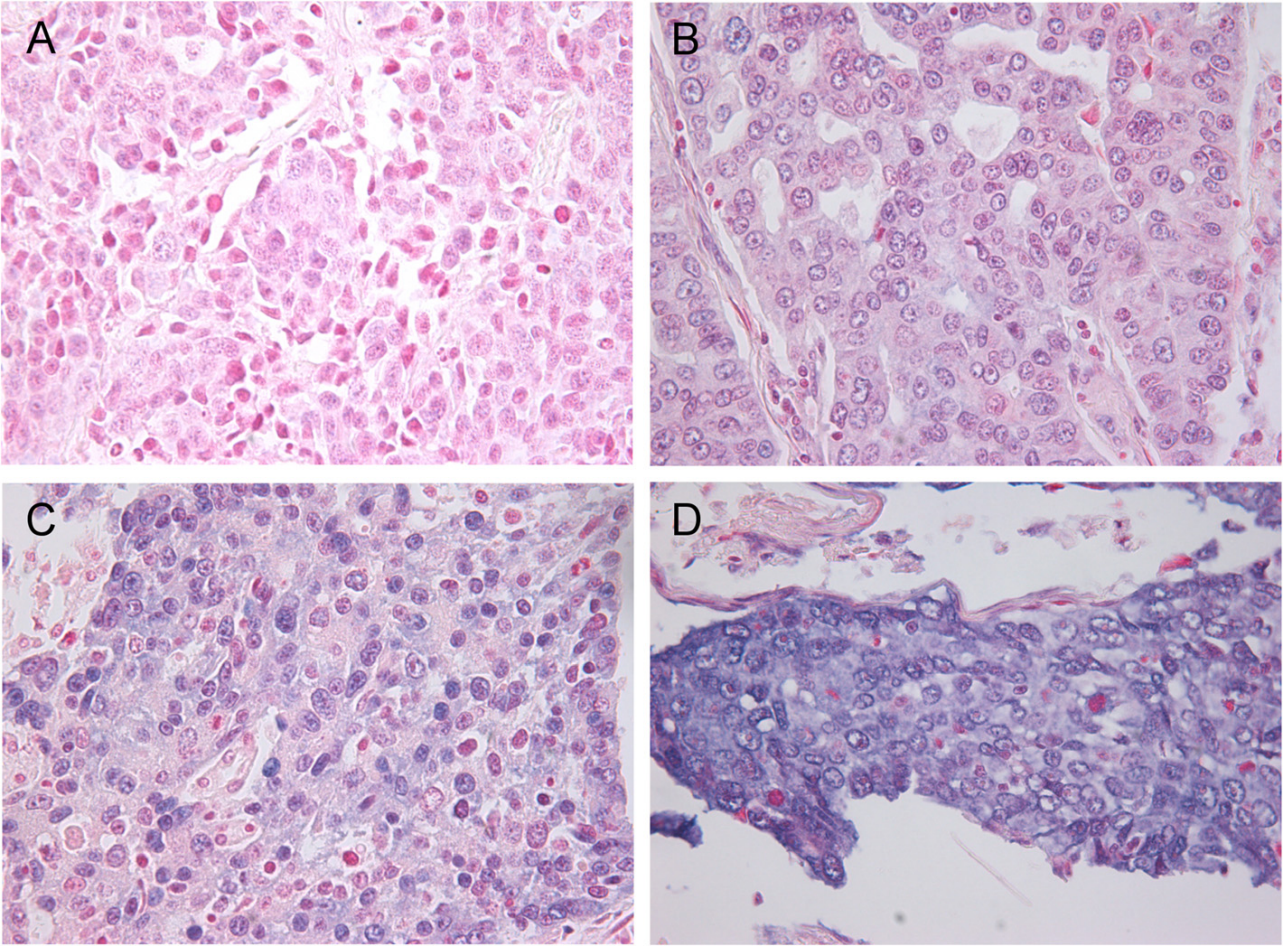
**In situ hybridization (ISH) analysis of non-small-cell lung cancer.** Scoring intensities based on blue cytoplasmatic staining graded from 0–3 in tumor cells. **A**: score 0; **B**: score 1; **C**: score 2; **D**; score 3.

Representative viable tissue sections were scored semi-quantitatively by light microscopy. The dominant staining intensity in tumor cells was scored as 0 = negative, 1 = weak, 2 = intermediate or 3 = strong (Figure 1). The TMA cores were scored anonymously and independently by one experienced pathologist and one oncologist. In case of disagreement, the slides were reexamined and consensus was reached by the observers.

Mean score for duplicate cores from each individual was calculated in tumor epithelial cells. We then categorized the staining into high and low expression. High expression in tumor cells was defined as score >0.

### Statistical methods

All statistical analyses were performed using the statistical package SPSS (Chicago, IL), version 19.0. The chi-square test and the Fisher exact test were used to examine the association between molecular marker expression and the clinicopathological markers. Correlations between markers were assessed using Spearman’s rank correlation. Univariate analyses were done using the Kaplan-Meier method, and statistical significance between survival curves was assessed by the log-rank test. Disease-specific survival (DSS) was defined as time from surgery to lung cancer death. Variables of significant value from the univariate analyses were entered into multivariate analysis using the backward stepwise Cox regression analysis. A P < 0.05 was considered statistically significant.

### Ethics

The National Data Inspection Board and the Regional Ethics Committee North (REC North) approved this study. Information and subsequent written consent from patients was considered, but as this was a retrospective study with more than half of patients deceased, the rest of the patients having to be reminded about the death rate of the disease and the possible raising of unrealistic hope for the individual, The Norwegian Data Inspection Board and REC North specifically waived the need for consent. All the patient data were anonymized after collecting the clinicopathological variables for each patient and before doing the statistical analyses.

## Results

### Patient characteristics

Demographic, clinical and histopathological variables are listed in Table 1. The median patient age was 67 (range 28–85) and the majority were male (76%). Most patients (95%) were current or previous smokers. The NSCLC tumors comprised 191 squamous cell carcinomas (SCC), 113 adenocarcinomas (AC) including 18 bronchioloalveolar carcinomas (BAC) and 31 large-cell carcinomas (LCC).

**Table 1:** Patient characteristics and their variables as predictors for disease-spesific survival in 335 NSCLC patients (univariate analyses; log-rank test)

**Characteristics**	**Patients**		**Median survival**	**5-year survival**	**P**
	**n**	**(%)**	**months**	**%**	
**Age**					
≤ 65 years	156	(47)	98	55	0.42
>65 years	179	(53)	NR	60	
**Sex**					
Female	82	(24)	190	64	0.22
Male	253	(76)	98	56	
**Smoking**					
Never	15	(5)	19	43	0.26
Current	215	(64)	NR	60	
Former	105	(31)	84	55	
**Performance status**					
PS 0	197	(59)	NR	63	**0.016**
PS 1	120	(36)	64	52	
PS 2	18	(5)	25	33	
**Weight loss**					
< 10%	303	(90)	190	58	0.76
> 10%	32	(10)	98	57	
**Histology**					
SCC	191	(57)	NR	66	**0.028**
Adenocarcinoma	113	(34)	54	46	
LCC	31	(9)	98	56	
**Differentiation**					
Poor	138	(41)	47	47	**< 0.001**
Moderate	144	(43)	190	65	
Well	53	(16)	NR	68	
**Surgical procedure**					
Lobectomy + wedge*	243	(73)	190	62	**0.007**
Pneumonectomy	92	(27)	37	47	
**Pathological stage**					
I	157	(47)	NR	61	**< 0.001**
II	136	(40)	62	51	
IIIa	42	(13)	17	23	
**Tumor status**					
1	85	(25)	190	75	**< 0.001**
2	188	(56)	84	57	
3	62	(19)	25	36	
**Nodal status**					
0	232	(69)	NR	67	**< 0.001**
1	76	(23)	35	43	
2	27	(8)	18	18	
**Surgical margins**					
Free	307	(92)	190	59	0.37
Not free	28	(8)	47	48	
**Vascular infiltration**					
No	284	(85)	190	62	**0.001**
Yes	51	(15)	27	33	

Statistically significant results in bold font.

*Wedge, n = 10.

*Abbreviations:NR* not reached, *PS* performance status, *SCC* squamos cell carcinoma, *LCC* large-cell carcinoma. Adenocarcinoma including cases with bronchioloalveolar carcinoma.

### Expression of miR-182 and correlations

miR-182 was homogenously expressed mainly in the cytoplasm of tumor cells. There was also some unspecific nuclear staining (Figure 1). The scoring was based on cytoplasmic staining. There was no staining of stromal cells, except for weak nuclear staining of some fibroblasts.

We tested correlations between miR-182 and angiogenic and hypoxia molecular markers. We found significant correlations between miR-182 and FGF2 (r = −0.147; P = 0.010), HIF2α (r = 0.115; P = 0.047) and MMP-7 (r = 0.172; P = 0.003).

### Univariate analysis

As shown in Table 1, the clinicopathological variables performance status (P = 0.016), histology (P = 0.028), tumor differentiation (P < 0.001), surgical procedure (P = 0.007), pathological stage (P < 0.001), tumor status (P < 0.001), nodal status (P < 0.001) and vascular infiltration (P = 0.001) were significant prognostic indicators for DSS.

**Figure 2:**
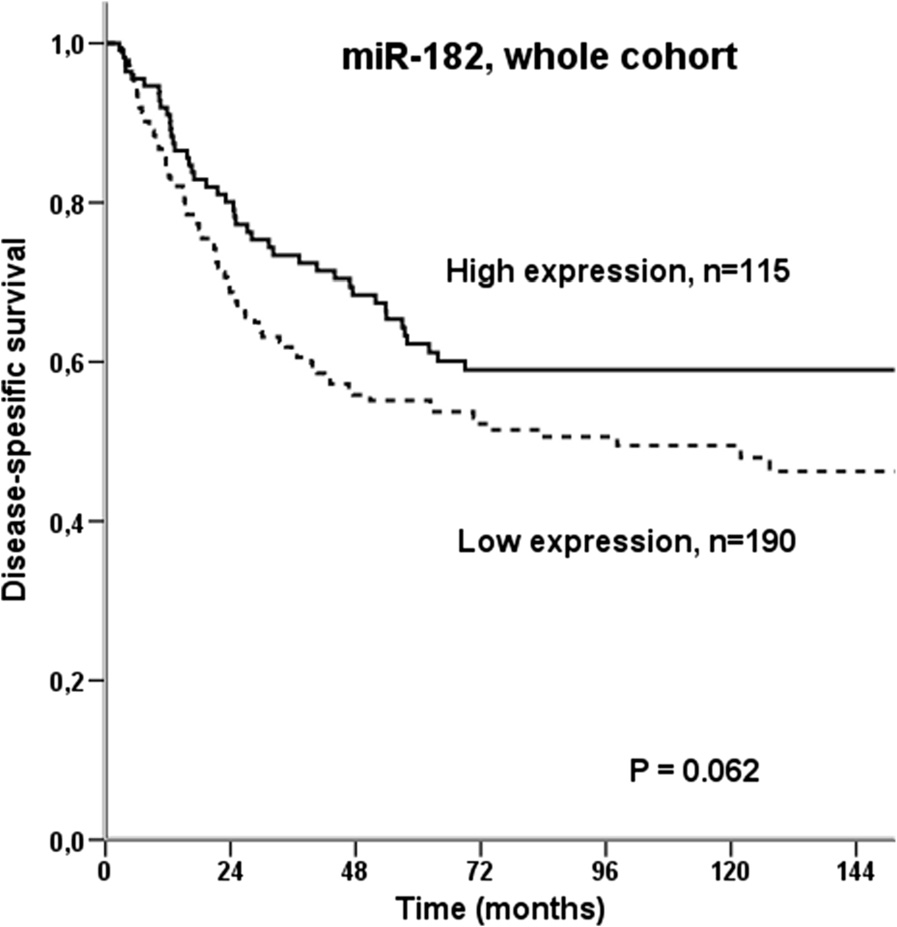
Disease-specific survival curves according to tumor cell expression of miR-182 in the whole cohort of patients.

**Figure 3:**
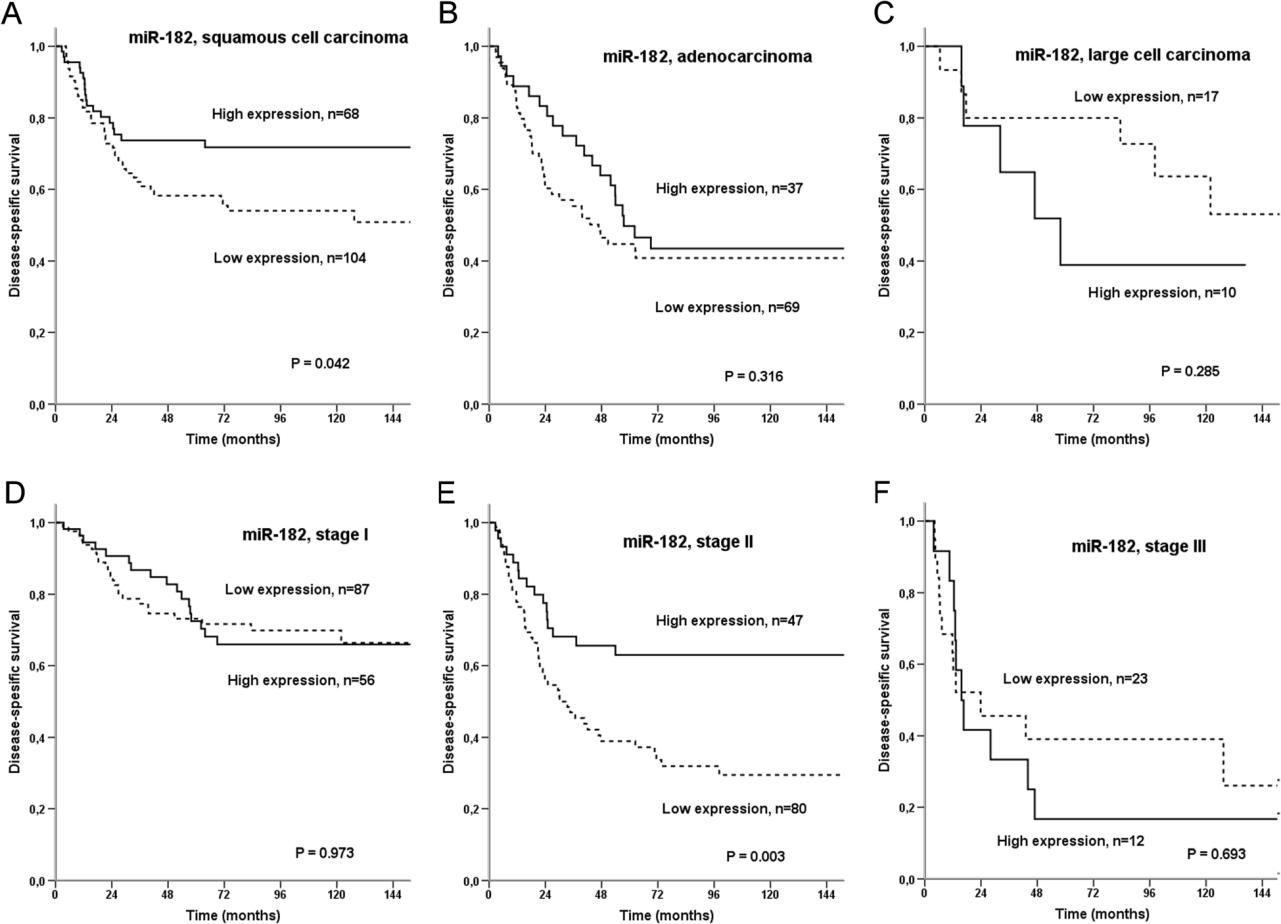
Disease-specific survival curves according to tumor cell expression of A) miR-182 in SCC, B) miR-182 in AC, C) miR-182 in LCC, D) miR-182 in stage I patients, E) miR-182 in stage II patients, F) miR-182 in stage III patients.

The results from the univariate analyses on miR-182 are presented in Table 2 and Figures 2 and 3. In the whole cohort, there was a tendency towards a better prognosis for those with tumors overexpressing miR-182 (P = 0.062, Figure 2). In subgroup analyses, patients with stage II disease had a significantly improved prognosis if they overexpressed miR-182 (P = 0.003, Figure 3E). In the histological subgroup SCC, high tumor cell miR-182 expression was associated with superior prognosis when compared to low expression (P = 0.042, Figure 3A), while for large cell carcinomas the trend was opposite (Figure 3C).

**Table 2:** miR-182 in tumor cells and stroma as predictors for disease-specific survival in NSCLC patients (univariate analysis; log-rank test) and results of Cox regression analysis summarizing significant independent prognostic factors

**Characteristics**	**Pts (n)**	**Pts (%)**	**Median survival (months)**	**5-year survival (%)**	**Univariate (P)**	**Multi-variate (P)**	**HR (95% CI)**
**Total (n = 335)**					0.062	0.098	0.73
Low	190	57	98	55			(0.50-1.06)
High	115	34	NR	62			
Missing	30	9					
**Pathological stage**							
**Stage I (n = 143)**					0.97	NE	NE
Low	87	61	190	73			
High	56	39	NR	73			
**Stage II (n = 127)**					**0.003**	**0.020**	**0.50**
Low	80	63	33	39			**0.28-0.90**
High	47	37	NR	63			
**Stage III (n = 35)**					0.69	NE	NE
Low	23	66	23	39			
High	12	34	15	17			
**Histology**							
**SCC (n = 172)**					**0.042**	**0.048**	**0.57**
Low	104	60	NR	58			**0.33-0.99**
High	68	40	NR	74			
**AC (n = 106)**					0.316	NE	NE
Low	69	65	47	45			
High	37	35	57	50			
**LCC (n = 27)**					0.285	NE	NE
Low	17	63	NR	80			
High	10	37	58	39			

Statistically significant results in bold font.

*Abbreviations:NR* not reached, *PS* performance status, *SCC* squamous cell carcinoma, *AC* adenocarcinoma, *LCC* large-cell carcinoma, *NE* not entered due to insignificance. Adenocarcinoma including cases with bronchioloalveolar carcinoma.

### Multivariate analysis

In the total cohort, performance status (P = 0.008), histology (P = 0.001), tumor differentiation (P = 0.007), tumor status (P = 0.007), nodal status (P = 0.022) and vascular infiltration (P = 0.004) all were independent prognostic factors.

Results of the multivariate analysis for miR-182 expression are presented in Table 2. Examining the total material, high miR-182 expression tended towards an independent association with a better prognosis (HR 0.73, CI 95% 0.50-1.06, P = 0.098). Among stage II patients, however, high tumor cell expression of miR-182 was an independent positive prognostic factor (HR 0.50, CI 95% 0.28-0.90, P = 0.020). Also in SCC, patients with a high miR-182 expression had an independent favorable outcome (HR 0.57, CI 95% 0.33-0.99, P = 0.048).

### Co-expression of miR-182 with FGF2 and MMP-7

**Figure 4:**
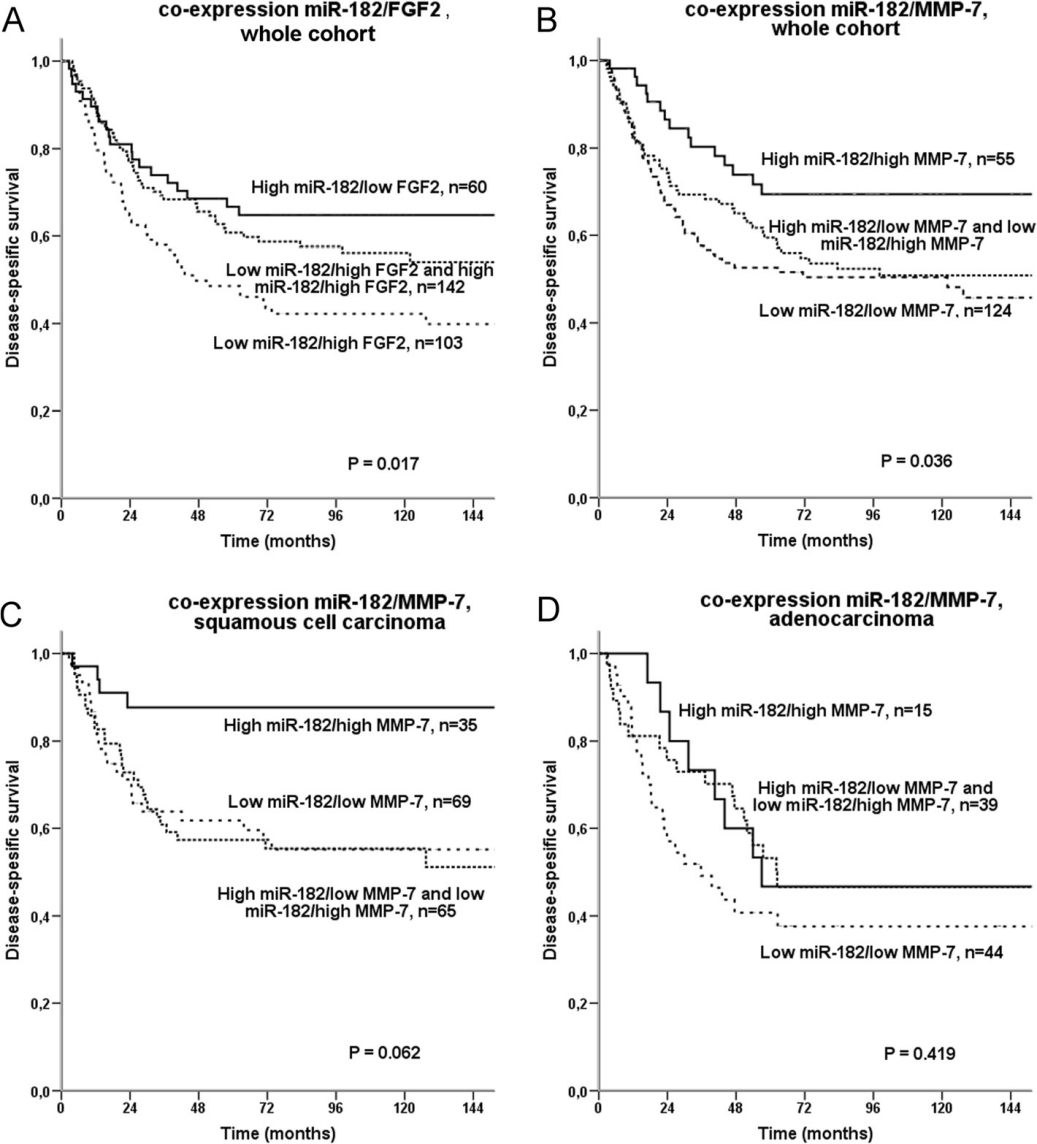
Disease-specific survival curves according to tumor cell co-expression of miR-182 and A) FGF2 in the whole cohort of patients, B) MMP-7 in the whole cohort of patients, C) MMP-7 in SCC, and D) MMP-7 in AC.

Among markers examined for correlations with miR-182, FGF2 and MMP-7 showed the strongest correlations. We assessed the co-expression combinations between miR-182 and FGF2 and MMP-7, respectively. The co-expression of low miR-182/high FGF2 was associated with poor survival (P = 0.017) as shown in Figure 4A. The combination showed an independently significant adverse prognosis compared to high miR-182/low FGF2 (HR 1.92, P = 0.015, Table 3). Patients expressing high miR-182/high MMP-7 had a better survival than other combinations (P = 0.036, Figure 4B). In the multivariate analyses, high miR-182/high MMP-7 showed an independently better prognosis than low miR-182/low MMP-7 (HR 0.49, P = 0.015, Table 3). In the SCC subgroup, we found an even bigger difference between these groups both in univariate and multivariate analyses (Figure 4C, Table 3).

**Table 3:** Results of Cox regression analysis summarizing co-expressions of miR-182 with FGF2 and MMP-7, respectively

	**Hazard ratio**	**95% CI**	**P**
**Co-expression of miR-182/FGF2**			**0.021**
High miR-182/low FGF2	1.00		
High miR-182/high FGF2 and low miR-182/low FGF2	1.26	0.74-2.13	0.39
Low miR-182/high FGF2	1.92	1.14-3.24	**0.015**
**Co-expression of miR-182/MMP-7**			**0.032**
Low miR-182/low MMP-7	1.00		
Low miR-182/high MMP-7 and high miR-182/low MMP-7	0.71	0.48-1.05	0.086
High miR-182/high MMP-7	0.49	0.27-0.87	**0.015**
**Co-expression of miR-182/MMP-7, squamous cell carcinoma**			**0.040**
Low miR-182/low MMP-7	1.00		
Low miR-182/high MMP-7 and high miR-182/low MMP-7	0.80	0.46-1.37	0.41
High miR-182/high MMP-7	0.26	0.090-0.74	**0.012**

Statistically significant results in bold font.

## Discussion

In a large unselected cohort of NSCLC patients we found miR-182 to be an independent positive prognostic factor in stage II patients and in patients with squamous cell carcinoma. We are, to our knowledge, the first group evaluating the prognostic impact of miR-182 in NSCLC using *in situ* hybridization.

Barshack and coworkers showed that miR-182 was over-expressed in primary lung tumors relative to metastases to the lung [24]. In another study by the same group, a set of different miRNAs could be used to differentiate hepatocellular carcinomas from metastatic tumors in the liver [25]. miRNA expression differs between tumor types, within the same tumor type in different patients and between primary tumors and metastases. Hence, it may not be surprising to find miR-182 to have divergent impact in different stages of NSCLC.

Increasing evidence demonstrate that adenocarcinomas and SCC of the lung are separate lung cancer entities, have dissimilar features and may respond differently to therapy. Targeted drugs with specific effects in certain histological subgroups have been developed. Certain miRNA-signatures can differentiate SCC from non-SCC and may facilitate the distinction between them [26]. Demonstrating a significant prognostic effect by miR-182 in SCC and not in adenocarcinomas underscores the diversity between the histological subgroups. In a previous published paper from our group [27], we explored the impact of miR-155 in the same cohort. We found this miRNA to be very stage- and tissue specific, with a significant impact on survival only in node positive SCC patients.

miR-182 has been regarded as an oncogene in most contexts. In a cohort of 253 glioma patients, high miR-182 expression was found to be a negative prognostic factor [12]. In melanoma cell lines, Segura and coworkers showed that high miR-182 expression stimulated migration and survival. The same group treated liver metastases in mice with anti-miR-182 and obtained a lower tumor burden and a lower mir-182-level than in untreated mice [13,28]. Also in breast tumors and cervical cancers miR-182 seems to have an oncogenic impact [29,30].

There are other studies that have identified miR-182 as a tumor suppressor. Kong et al. found miR-182 to be underexpressed in human gastric cancer cell lines. They showed that the oncogene cAMP responsive element binding protein 1 (CREB1) is a target of miR-182, and that high levels of miR-182 leads to lower levels of CREB1 and suppressed gastric adenocarcinoma cell growth [31]. In melanoma cell lines, Poell et al. found miR-182 to be a strong inhibitor of cell proliferation [14]. Yan and coworkers found similar effects in uveal melanoma cells, where they identified MITF, BCL2 and cyclin D2 as potential targets of miR-182. Transfection of miR-182 into cultured uveal melanoma cells led to a significant decrease in cell growth, migration and invasiveness [16].

In lung cancer, data on miR-182 have been conflicting regarding its prognostic role. In 70 lung cancer tissue samples, Zhu and coworkers observed an association between high expression of the members of the miR-183 family (miR-96, miR-182 and miR-183) and poor overall survival [11]. In contrast, two in vitro studies using cell lines did not support the notion of miR-182 exerting an oncogene role in lung cancer. Sun et al. found miR-182, through regulation of RGS17, to suppresses lung tumorigenesis [15]. Consistently, Zhang and coworkers reported miR-182 to inhibit proliferation and invasion of human lung adenocarcinoma cells via its effect on human cortical actin-associated protein (CTTN) [32].

miR-182 has a number of target genes, and it is evident that the regulation of these genes can result in both inhibition and stimulation of tumorigenesis. In NSCLC, our results suggest that tumor inhibiting miR-182 features dominate and thus make this miRNA a favorable prognostic factor.

Based on the association with angiogenesis suggested from the GSEA [17], we investigated the correlation between miR-182 and a set of angiogenesis-related protein markers. There was a negative correlation between miR-182 and FGF2. Our group has published data on FGF2, which identify this marker as an independent negative prognostic factor in lung cancer cells [22]. Fibroblast growth factor receptor substrate 2 (FRS2) is a downstream mediator of the fibroblast growth factor pathway and is a target gene of miR-182. FRS2 is thought to induce tumor progression through stimulation of angiogenesis [17,33]. In our total NSCLC cohort, the coexpression between miR-182 and FGF2 showed an independent significantly worse prognosis for low miR-182/high FGF2 than for high miR-182/low FGF2 (P = 0.015, Table 3).

A correlation was also detected between miR-182 and MMP-7. In a previous paper, our group found high MMP-7 expression to be an independent favorable prognostic factor in this same NSCLC cohort [23]. When examining coexpression of the two variables, those with high miR-182 and high MMP-7 expression had an independently better survival than those with low miR-182/low MMP-7 expression (HR 0.49, P = 0.015). When stratifying on histology, the SCC patients with high/high expression had a remarkably better prognosis than the rest of the groups (HR 0.26, P = 0.012, Table 3).

To our knowledge, there are no published data linking miR-182 and MMP-7. Few studies have described the connection between FGF2 and MMP-7 [34,35]. Based on our strong results from the co-variations between miR-182 and particularly MMP-7, it would be interesting to see functional studies exploring potential relations between these two markers.

In our previous pilot study on miRNA signatures [17], miR-182 appeared as an oncogene since it was up-regulated in short vs long term NSCLC survivors and in NSCLC vs normal tissues. In our large unselected NSCLC cohort presented herein, we surprisingly observed that high miR-182 expression is associated with improved survival, at least in subgroups of patients with NSCLC. It has to be kept in mind that the explorative study was based on a small sample, only 20 NSCLC cases and 10 normal lung tissues. Hence, the contrasting results may be due, at least in part, to selection bias in the explorative study. Besides, in the present study the favorable prognostic impact by miR-182 was seen in subgroups of NSCLC patients, and assessments were tissue specific (only in tumor cells) using *in situ* hybridization and not real time qPCR, as in the pilot study [17]. When using qPCR a contribution from the stromal compartment will influence the result, and the stromal expression of miR-182 may be different from that of the tumor cells.

## Conclusion

In conclusion, miR-182 tended to be a favorable prognostic factor in the total NSCLC cohort. Moreover, in stage II and in SCC patients we found miR-182 to have tumor suppressor properties. Nevertheless, our study must be regarded as hypotheses generating, and needs to be confirmed in other cohorts and functional studies. We found a weak, but significant association between mir-182 and the angiogenesis related markers FGF2 and MMP-7. It would be interesting to see further studies exploring these associations.

## Competing interests

The authors declare that they have no competing interests.

## Authors’ contributions

HS participated in the design of the study, contributed to the clinical and demographic database, did the statistical analysis and drafted the manuscript. TD, SA and SAS contributed to the clinical and demographic database and SAS in making the TMAs. TD and SA contributed to the statistical analysis. SAS and HS scored the cores. RMB and LTB supervised and participated in the study design, result interpretation and writing. All authors read and approved the final manuscript.

## Pre-publication history

The pre-publication history for this paper can be accessed here:

http://www.biomedcentral.com/1471-2407/14/138/prepub
